# Health informatics standardization and RHIS construction patterns

**DOI:** 10.1186/1479-5876-10-S2-A54

**Published:** 2012-10-17

**Authors:** Guanhua Ren

**Affiliations:** 1 China national institute of standardization, Beijing, 100088, China

## 

This report mainly introduces the importance of health informatics standardization and the current international and Chinese research status, analyzes the developing trend and prospects of health informatics standardization, discusses the RHIS (regional health information system) of two kinds of construction Patterns. On this basis, this report points out the difficulties of health informatics standardization implementation and application, and some corresponding resolving measures and advice.

